# 3D *In Vitro* Models of the Bone Marrow Niche

**DOI:** 10.1021/acsbiomaterials.5c01421

**Published:** 2025-12-12

**Authors:** Pasqualina Scala, Bianca Serio, Valentina Giudice

**Affiliations:** † Department of Medicine, Surgery, and Dentistry, University of Salerno, Baronissi 84081, Italy; ‡ Hematology and Transplant Center, University Hospital “San Giovanni di Dio e Ruggi d’Aragona”, Salerno 84131, Italy

**Keywords:** 3D cultures, bone marrow, niche, tissue engineering, scaffolds

## Abstract

The bone marrow niche is a specialized microenvironment sustaining a hematopoietic stem cell (HSC) pool and regulating the production of mature blood cells. Its exact composition and mechanisms remain incompletely defined, mainly due to the lack of *in vitro* models that accurately reproduce its physiological three-dimensional (3D) architecture and cellular crosstalk. Two-dimensional cultures fail to sustain HSC quiescence and stemness, while advanced 3D systems can reproduce key structural and mechanism cues of the niche. In this review, we first describe physiological cellular, stromal, and matrix components of the bone marrow niche, highlighting their coordinated regulation of HSC maintenance, proliferation, and mobilization. We then critically examine current approaches for 3D *in vitro* bone marrow models, including scaffold-based methods, decellularized models, spheroid and organoid systems, 3D bioprinting applications, and organ-on-chip technologies, discussing their advances, limitations, and potential disease modeling in this field. Finally, we outline how these technologies could deepen our understanding of hematopoiesis mechanisms, clonal evolution, and niche-mediated drug resistance. We also highlight the pros and cons of each methodology and future directions toward standardized protocols, integrating tissue components, and the use of human cells to enhance reproducibility and clinical relevance. Advances like bone marrow-on-a-chip, computational models, and patient-specific systems will help bridge the gap between *in vitro* and *in vivo* studies, enabling drug testing, stem cell expansion, and gene editing strategies, including chimeric antigen receptor expression. Bone marrow models have evolved from simple 2D cultures to advanced 3D and organ-on-a-chip systems, significantly improving our understanding of hematopoiesis and accelerating new therapies.

## Graphical abstract

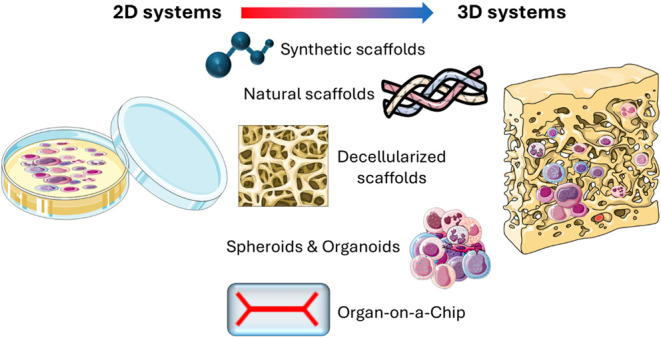

## 1 Introduction

Hematopoiesis is the physiological process by which blood components are produced, to sustain oxygen/nutrients exchange, immune responses, and hemostasis and coagulation.^1^ In adulthood, it persists only within certain bones, like flat bones and the ends of long bones, while in other places it is replaced by adipose tissue.^1^ The bone tissue forms a structured support for hematopoietic stem and progenitor cells (HSPCs), where they can proliferate and differentiate, surrounded by other cellular components, such as mesenchymal stem cells (MSCs), bone marrow adipocytes (BMAd), fibroblasts, macrophages, endothelial cells, vessels, and also by other noncellular components, like extracellular matrix, cytokines, and growth factors.^2^ A complex crosstalk between all these components finely regulates the hematopoiesis, and this intricated, and mostly uncharacterized system is known as bone marrow hematopoietic niche.^3^ The term “niche” refers to a local microenvironment involved in the maintenance and regulation of specific stem or progenitor cells.^4^ In the bone marrow, hematopoietic stem cells (HSCs) reside next to osteoblasts in the endosseous niche or adjacent to endothelial cells in sinusoidal vessels, protected areas where HSCs are retained in close contact with stromal cells through several homing signals, such as those triggered by stromal cell-derived factor 1 (SDF-1), also known as CXC motif chemokine 12 (CXCL12), and C-X-C chemokine receptor type 4 (CXCR4).^2,5^ Under physiological conditions, mature cells leave the bone marrow in response to changes in SDF-1 levels and are released into the bloodstream; conversely, HSC homing occurs in response to higher levels of SDF-1 in the bone marrow.^6^ The osteoblastic niche provides a quiescent microenvironment for HSC maintenance, while the vascular niche favors transendothelial migration and HSC proliferation and differentiation, through endothelium-derived fibroblast growth factor 4 (FGF4) and SDF-1 signaling.^7^ Higher gradients of FGF4 and oxygen concentration observed as cells progress from the osteoblastic niche to the vascular niche could play a role in the recruitment, proliferation, and differentiation of HSCs/HSPCs.^8,9^ Under stress, SDF-1 and vascular endothelial growth factor (VEGF) activate matrix metalloproteinase-9 (MMP-9), which converts the membrane-associated Kit ligand into soluble Kit ligand (sKitL) and in turn promotes the entry of HSPCs into the cell cycle, mobilization in the vascular niche, and differentiation.^10^ Moreover, in malignant diseases, bone marrow niche architecture and composition are completely overturned, with vascular remodeling, altered microenvironment composition in terms of different concentrations of cytokines, chemokines, growth factors, impaired adhesion, and metabolic switch that enhances adaptability to hypoxia and oxidative stress.^11^

*In vitro* maintenance of HSCs is challenging because stem cell survival and differentiation strictly depend on cell-to-cell contact and paracrine signals, which are spatially organized in different bone marrow areas, to ensure stemness maintenance or promote mature cell differentiation. For these reasons, *in vitro* reproduction of these complex cross-talks and microarchitecture is extremely difficult.^12^ 3D-culture systems, such as sodium and calcium alginate hydrogels, effectively replicate tissue structure and support the expansion of CD34^+^CD38^–^ stem and CD34^+^CD38^+^ progenitor cells using various bioengineered scaffolds.^13,14^ Advances in culture medium composition have improved long-term *in vitro* maintenance of hematopoietic precursors, such as by including basic fibroblast growth factor (bFGF) together with dexamethasone, ascorbic acid, and β-glycerophosphatein in rat stromal bone marrow cell culture for mineralized bone-like tissue formation.^15^ Other cytokines and growth factors usually added to culture medium are interleukin (IL)-3, granulocyte-macrophage colony-stimulating factor (GM-CSF), erythropoietin (EPO), stem cell factor (SCF), IL-16, thrombopoietin (TPO), and FMS-like tyrosine kinase 3 (FLT3)-ligand.^16,17^ There are additional compounds with the ability to preserve stemness, such as stem regenin-1 and 16,16-dimethyl prostaglandin E2;^18^ however, the implementation of highly mimicking bone tissue systems would eventually lead to no further need for medium supplementation, as soluble factors would be released by fabricated supportive models.^19^

In this review, we summarize the physiological composition of the bone marrow niche and current 3D *in vitro* models used in research and in regenerative medicine, aiming to reproduce normal interactions and tissue structures, mimic the BM niche in health and diseases, and better understand the pathophysiology of normal hematopoiesis and hematological malignancies.

## 2 Bone Marrow Niche Composition

The bone marrow niche is divided into two interconnected regions: the vascular and the osteoblastic niche (Figure 1).^20^

**Figure 1:**
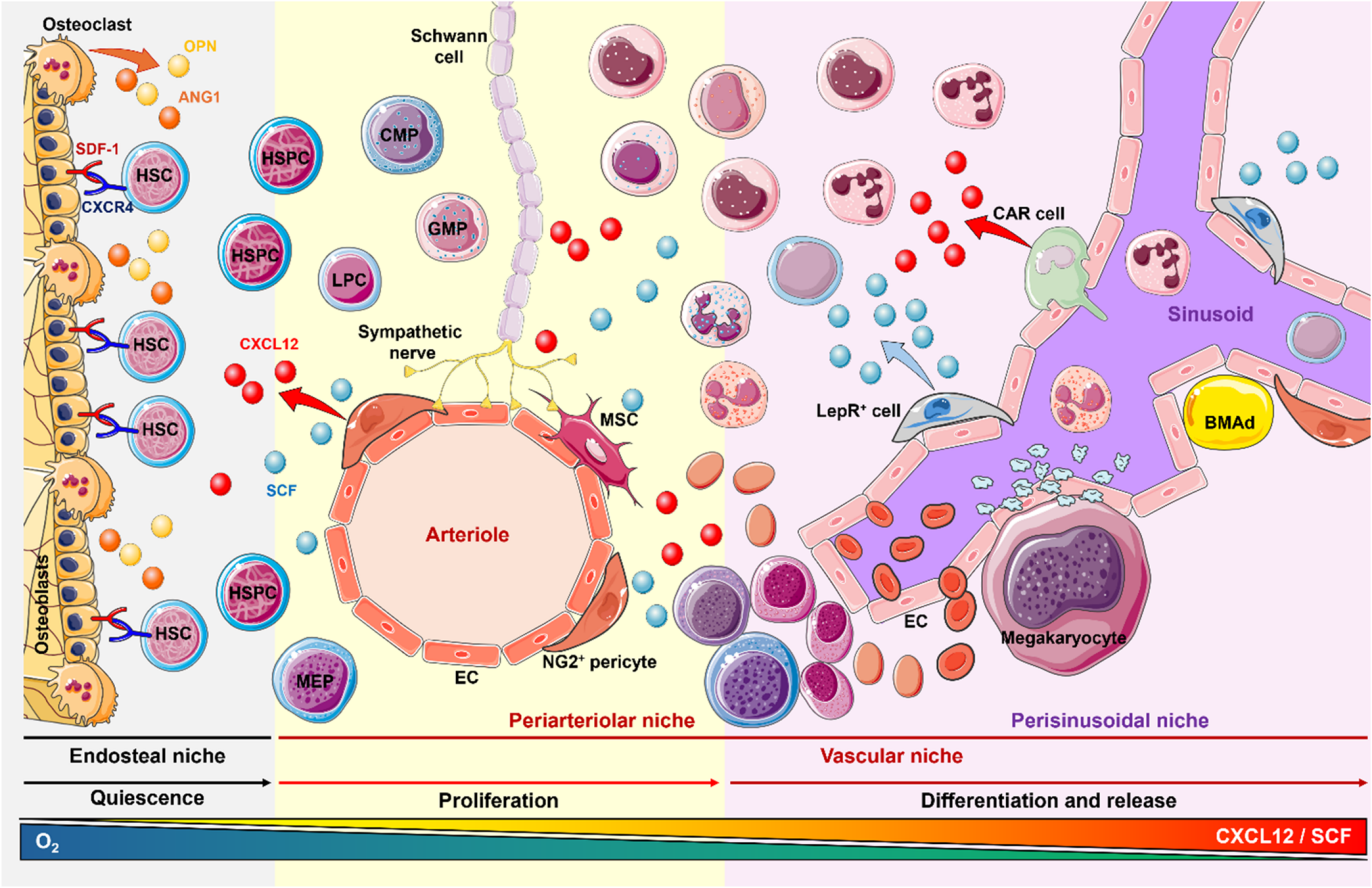
Bone marrow niche composition. The bone marrow niche is mainly divided into three areas: endosteal, periarteriolar, and perisinusoidal niche. Hematopoietic stem cells (HSCs) are retained in the endosteal niche through CXCR4-SDF-1 interaction and remain quiescent. Perivascular stromal cells, including endothelial cells (EC), neuron-glial antigen 2 (NG2+) pericytes, bone marrow adipocytes (BMAd), and mesenchymal stem cells (MSCs), regulate HSC proliferation via secretion of CXC motif chemokine ligand 12 (CXCL12), also known as stromal cell-derived factor 1 (SDF-1), and stem cell factor (SCF). The production of these factors is also influenced by the nervous system through sympathetic innervations in the bone marrow. Once released from the endosteal niche, under the CXCL12/SCF gradient, HSCs start to proliferate and differentiate, until mature cells are not completely formed and released into the bloodstream. CAR cells, Nestin+ cells, and Leptin Receptor (LepR)+ cells, located near the sinusoids, contribute to the production of CXCL12 and SCF, by increasing their gradient as close as mature cells are to sinusoids and are ready to be released. Created using https://smart.servier.com/.

### 2.1 Vascular Niche

Endothelial cells (ECs) and perivascular stromal cells, including pericytes and smooth muscle cells, compose the blood vessels of the vascular niche,^21^ that function as structural support and secrete essential factors for HSC maintenance, proliferation, and differentiation.^22^ ECs originate from hemangioblasts, multipotent progenitor cells present in the embryonic stage, from which HSCs also originate.^23,24^ RUNX1-expressing endothelial cells can generate HSCs within the aorta, gonad, mesonephros, and placenta.^25^ In turn, HSCs can secrete angiopoietin-1 (ANG1), which promotes new blood vessel formation during angiogenesis.^26^ Endothelial and HSCs commonly express CD31, CD34, CD133, FLK1, and TIE2.^27^ ECs, located at the interior side of blood vessels, have a distinctive Notch^+^SDF-1^+^SCF^+^VEGFR2^+^pleiotrophin^+^ phenotype, highly influence HSC metabolism and cell cycle,^28^ participate in forming the vascular niche for HSCs and long-term HSCs, and favor HSC engraftment.^29^ HSCs usually localize in proximity of sinusoidal SCF-producing ECs and of arteriolar capillary-forming cells within the endothelium, which can also secrete a developmental endothelial locus-1, a glycoprotein that promotes HSC proliferation and myeloid lineage differentiation.^30,31^

The vascular niche also regulates cell cycle and quiescence, as quiescent HSCs and >80% of long-term repopulating HSPCs are found close to sinusoids, where they interact with LepR^+^SDF-1^high^ cells and endothelial cells.^2,32,33^ Cell cycle entry can be promoted by endothelial progenitor cells through MMP-9 activation in osteoblast regions and by the release of sKitL, which stimulates HSC activity in the vascular niche.^34^ Quiescent HSCs start to differentiate upon SDF-1 and SCF stimulation, secreted by Nestin^+^ cells around the arterioles, pericytes, smooth muscle cells, LepR^+^ cells, and SDF-1-abundant reticular (CAR) cells. Angiogenic factors, such as VEGF and ANG1, are also vital for HSC maintenance, as VEGF influences vascular development and hematopoiesis.^35^

HSCs remain dormant in osteoblastic niches and migrate to the vascular areas for proliferation and differentiation.^36^ For this reason, several homing factors and gradients are essential to regulate the HSC exchange between these two niches.^37^ SDF-1 is important for HSC homing, as its knockout results in the removal of quiescent and transplantable HSCs by disrupting CXCR4 signaling.^38,39^ An increasing SDF-1 gradient allows HSC mobilization from the periphery toward areas with higher SDF-1 levels, favoring cell differentiation.^40^ Moreover, SDF-1 induces the secretion of VEGF and metalloproteinases, leading to neoangiogenesis and remodulation of oxygen distribution.^41^ Indeed, oxygen gradient modulates HSC quiescence, proliferation, and localization, as quiescent cells are more frequently enriched near arteriole-rich endosteal regions, while under stress (e.g., chemotherapy), the endosteal niche supports HSC quiescence and maintenance.^42,43^ Upon stress-induced activation, SCF is released, and HSCs start to migrate from the perisinusoidal space to the endothelial cells, where they adhere and communicate with parenchymal cells.^44^ Under physiological conditions, the bone marrow niche is a hypoxic tissue, with an approximate oxygen tension of 7–43 mmHg (<1–6% of oxygen saturation), and it decreases from the vascular to the endosteal niche.^45^ Granulocyte colony-stimulating factor (G-CSF) and cyclophosphamide can further extend this hypoxic region and subsequently regulate hypoxia-inducible factor (HIF) factors.^46^ HIF1A and HIF2A subunit expression is modulated by oxygen levels, inducing stabilization as heterodimers under hypoxic conditions, leading to binding to Hypoxia-response elements in the nucleus and transcription of genes, such as VEGF, erythropoietin, glucose transporters, and anaerobic glycolytic enzymes.^47^

### 2.2 Endosteal Niche

The endosteal or osteoblastic niche is composed of spongy bone, osteoblasts, osteoclasts, spindle-shaped N-cadherin^+^ osteoblasts (SNO) cells, arterioles, and sinusoids, as it is highly vascularized and favors HSC quiescence, especially long-term stem cells.^6,48^ Approximately 20–30% of quiescent HSCs are in close contact with endosteal osteoblasts, while rapidly cycling stem cells with blood vessels in the subendosteal region, where osteoblasts reside and contribute to HSC maintenance and self-renewal.^22,49^ This spatial heterogeneity of HSC compartment has been confirmed using different methods, such as cellular uptake of the fluorescent dye Ho in *in vivo* perfusion or whole-mount confocal immunofluorescence imaging techniques combined with computational modeling using mouse bone marrow tissues, also showing that arteriolar niche cells remain quiescent and are also protected from myeloablation and aging.^50–53^ Indeed, arteriolar HSCs proliferate less after cytotoxic treatment with 5-fluorouracil, while cells expand more at further distances from Nestin^+^ perisinusoidal cells.^54^

Osteoblasts arise from multipotent MSCs, while osteoclasts arise from CD34^+^ HSCs; however, endothelial progenitors can also become osteoblasts.^55^ These cells express G-CSF and influence HSC maintenance and trafficking via NOTCH, SDF-1, ANG1, and osteopontin. SDF-1/CXCR4 signaling is crucial for HSC mobilization, while osteopontin regulates HSC pool size and egress.^56^ Additionally, osteoblasts contribute to T lymphopoiesis via DLL4, which supports thymic progenitor development. Their depletion dramatically reduces the early B cell populations. Osteopontin and ANG1 help maintain HSC quiescence and prevent premature activation or migration.^57^ Indeed, HSCs closest to osteoblasts tend to remain dormant, and N-cadherin+ osteoblasts tend to form nests around HSCs, which slowly cycle HSCs.^2^ Osteopontin acts as “glue”, as thrombin cleaves the osteopontin fragment, creating an adhesive substrate for HSCs and exposing the binding site for integrin alpha9beta1 on HSCs.^58^

Osteoclasts originate from monocyte-macrophage lineage under specific factors, like receptor for nuclear factor-kappa B ligand (RANKL) and macrophage colony-stimulating factor (M-CSF), and play a role in bone resorption and HSC mobilization.^59^ Moreover, bone marrow CD169^+^ macrophages can maintain HSC quiescence and niche residency, especially through SDF-1 signaling.^60^ Osteomacs, a subset of macrophages close to osteoblasts, help regulate osteoblast function via nuclear factor κB (NF-κB) signaling, supporting HSC maintenance. Cooperation among osteomacs, osteoblasts, and megakaryocytes further shapes HSC repopulation capacity.^61^ Osteoblasts also finely tune osteoclast differentiation through M-CSF and RANKL, which are also expressed by bone marrow-associated stromal cells.^62^

Bone marrow-derived mesenchymal stem cells (BM-MSCs) modulate HSC functions through paracrine and cell-to-cell contacts and by acting as a reservoir for growth factors and drugs.^63^ MSCs show different immunophenotypes and abilities based on where they localize in the bone marrow niche.^64^ In the perivascular regions, MSCs express SDF-1, Leptin receptor (LepR), Neuron-glial antigen 2 (NG2), CD146, and Nestin, while in the endosteal niche, mesenchymal cells are more differentiated toward osteogenic precursors and are CD146^–^ cells. LepR^+^ MSCs can differentiate into osteolineage cells, and express various osteoblast-associated genes, such as Runx2, alkaline phosphatase, osteopontin, and osteocalcin, and some cells can also express SDF-1.^32^ LepR^+^ MSC osteoprogenitors usually locate around peri- and trabecular bone tissue and produce collagen type I (COL1).^55^ In the endosteal region, N-cadherin^+^CD45^–^ osteoblasts regulate HSC quiescence by cell-to-cell contacts and also via ANG1 and its receptor and through interaction with osteoblasts, SDF-1^+^ reticular cells, Nestin^+^ MSCs, Schwann cells, and perivascular cells.^65^ Nestin^+^ MSCs coexpressing NG2 are located around periarteriolar niches, have low SDF-1 and SCF expression, and slightly affect HSC numbers.^66^ Conversely, the nervous system tightly regulates hematopoiesis, as sympathetic nerve terminals control circadian HSC release through noradrenaline, modulation of SDF-1 expression in Nestin^+^NG2^+^ perivascular MSCs, while Schwann cells influence resting HSCs via tumor growth factor (TGF)-β/SMAD signaling.^67^ Adrenergic signaling via β3-adrenergic receptors leads to downregulation of SDF-1, and Nestin^+^ BMSCs and adrenergic nerve fibers are highly interconnected, influencing HSC functions and mobilization.^68^ CAR cells derive from MSCs, highly produce SDF-1, and closely interact with HSCs near sinusoids.^69^

BMAds are among the most abundant mesenchymal cells in the BM, and contribute to regulating bone metabolism and hematopoiesis, as they are now recognized not only as inert space fillers but also as critical regulators of various cellular and molecular mechanisms, such as regulation of hematopoiesis, secretion of adipokines, bone remodeling, and glucose and lipid homeostasis.^70^ BMAds significantly influence both endosteal and vascular niches, as BMAds contribute to bone resorption by secreting adipokines and enhancing RANKL and tumor necrosis factor (TNF)-α expression, which is accelerated during aging as aged cells secrete senescence-associated secretory phenotype factors and upregulate RANKL and TNF-α. Moreover, free fatty acids and adiponectin modulate SDF-1, SCF, and Fe ion availability, thus maintaining HSC homeostasis. Within the vascular niche, adult BMAds negatively influence neoangiogenesis through type H vessel decline, decreased RUNX2 expression, and osteoblast activity.^71^

### 2.3 Extracellular Matrix

The bone marrow extracellular matrix surrounds, supports, and regulates hematopoiesis and is composed of various collagens, fibronectin, laminin, and proteoglycans.^72,73^ Laminin and fibronectin offer structural support for HSCs and progenitors, while cell adhesion molecules, such as cadherins, selectins, members of the immunoglobulin superfamily, and integrins, modulate HSC functions and mobilization through cell-matrix interactions and signal transduction, acting as mechanoreceptors.^74^ Endothelial cells and BMAds promote basement membrane formation. Extracellular matrix (ECM) acts as a “growth factor net”, as matrix molecules can bind growth factors, increasing their local concentrations and facilitating cellular interactions. Moreover, stromal cells secrete active molecules, like SDF-1, ANG1, and SCF-1, which are included in the bone marrow matrix.^72,75^ Fibronectin is present in the extracellular matrix as an insoluble homodimer composed of two chains linked by a disulfide bond and consisting of repeating type I, II, or III domains, that interact with integrin receptors, integrins, heparin, proteoglycans, and collagens, facilitating extracellular matrix cross-linking.^76^ Dimeric fibronectin is abundantly produced by BM-MSCs, and regulates erythroid cell differentiation after erythropoietin stimulation, as well as megakaryocyte and platelet differentiation.^77^

Various collagen types are present in the bone marrow, including COL1, COL3, COL4, COL5, COL6, and COL14.^78^ COL1 is the most abundant collagen in the bone marrow matrix and is produced by osteoblasts and stromal cells. COL1 supports HSC adhesion and influences their quiescence, while also promoting MSC proliferation and osteogenic differentiation.^79^ COL3 is prevalent near arterioles and periosteal areas as sporadic fibrils and facilitates bone development.^72^ COL4 is present in endosteal, periarteriolar, and sinusoidal areas, influences prothrombocytes differentiation, and is expressed on megakaryocytes.^78^

Perlecan, a heparan sulfate proteoglycan, and hyaluronic acid, a glycosaminoglycan, are the most important proteoglycans in the bone marrow matrix. Perlecan is produced by MSCs and contributes to the internal connective tissue architecture and has antiadhesive properties toward HSCs. Moreover, perlecan can bind GM-CSF and regulate HSC differentiation.^80^

## 3 3D Models

Bone marrow niche is a complex and variegated microenvironment, where hematopoiesis is finely tuned through cell-to-cell contacts, paracrine signals, growth factors, cytokines, and chemokines, and cell type composition of surrounding areas.^2^ Most of these interactions are still unknown, and *in vitro* reproduction of these cross-talks is challenging. 3-dimensional (3D) *in vitro* models can better mimic physiological tissue architecture, thus overcoming issues related to standard 2D cultures.^13^ For bone marrow cells, HSCs quickly differentiate into 2D conditions and lose their stemness because of the lack of direct contact to stromal cells, as well as paracrine and growth factor signaling.^81^ Using 3D models, it is possible to coculture different cell types at the same time and to reproduce a porous environment resembling the trabecular bone regions where bone marrow components are hosted. Moreover, 3D models can be useful to mimic *in vitro* hematological diseases and their related bone marrow niche impairment.^82^ Indeed, the entire bone marrow niche contributes to disease development, as for example, osteoblasts are involved in preleukemic conditions in mice through activating mutation of β-catenin, which stimulates Notch ligand jagged 1 expression in osteoblasts and induction of Notch signaling in HSCs, thus promoting neoplastic transformation. Increased β-catenin expression in osteoblasts has been detected in 38% of patients with myelodysplastic syndromes or acute myeloid leukemia.^83^ Moreover, leukemic cells trigger bone morphogenetic protein 2 (BMP-2) and SMAD1/5 mechanisms in MSCs, inducing osteogenesis within a leukemogenic microenvironment.^84^ In addition, leukemic cells directly communicate with MSCs and form connexin-43-based gap junctions, which are used by neoplastic cells to reprogram MSCs toward a pro-oncogenic phenotype.^85^ In 2D culture, leukemic cells spontaneously trigger apoptosis, because of the lack of these interactions between neoplastic cells and the bone marrow microenvironment.^86,87^ Early bone marrow niche models were reproduced in 2D culture, where freshly isolated CD34^+^CD33^+^ cells were cultured in growth factor-supplemented media. Although 2D culture has high efficiency and low costs, the lack of cell–cell and cell-environment interactions induces rapid differentiation and stemness exhaustion, with a high apoptotic rate.^88^ 3D cultures are gaining importance in tissue engineering and regenerative medicine, because of the possibility to culture multiple cell populations and to mimic physiological tissue architecture, for reproducing stem cell niches, such as the satellite cell niche in muscle cell regeneration or the bone marrow niche.^89^ 3D *in vitro* models are divided based on the methodology used to embed cells and stroma, including scaffold-free and scaffold-based approaches. However, each of these approaches has pros and cons, as scaffold-free systems using bioreactors allow cell interactions but show limited HSC expansion, or hypoxic hydrogels can completely abolish HSC proliferation.^90^ Moreover, the source of MSCs and HSCs can influence the reliability and reproducibility of the 3D *in vitro* system. Indeed, MSCs and HSCs can be obtained from various sources, such as bone marrow, peripheral blood, and umbilical cord; however, limited studies directly compare the expansion and differentiation properties of stem cells of different origins and culture medium composition and conditions. Therefore, each source has its advantages and disadvantages. For example, peripheral blood-derived stem cells are easier to collect compared to bone marrow-derived stem cells, although the number of circulating cells is lower (higher only in G-CSF-stimulated subjects), similar to umbilical cord blood-derived stem cells, where the number of cells is limited due to the small blood volume obtained. However, differentiation potential is comparable between sources, while proliferation capacity is higher in bone marrow- and umbilical cord blood-derived stem cells.^91^

Recent advancements in 3D *in vitro* bone marrow models are summarized in Tables 1–2 and Figure 2.

**Figure 2:**
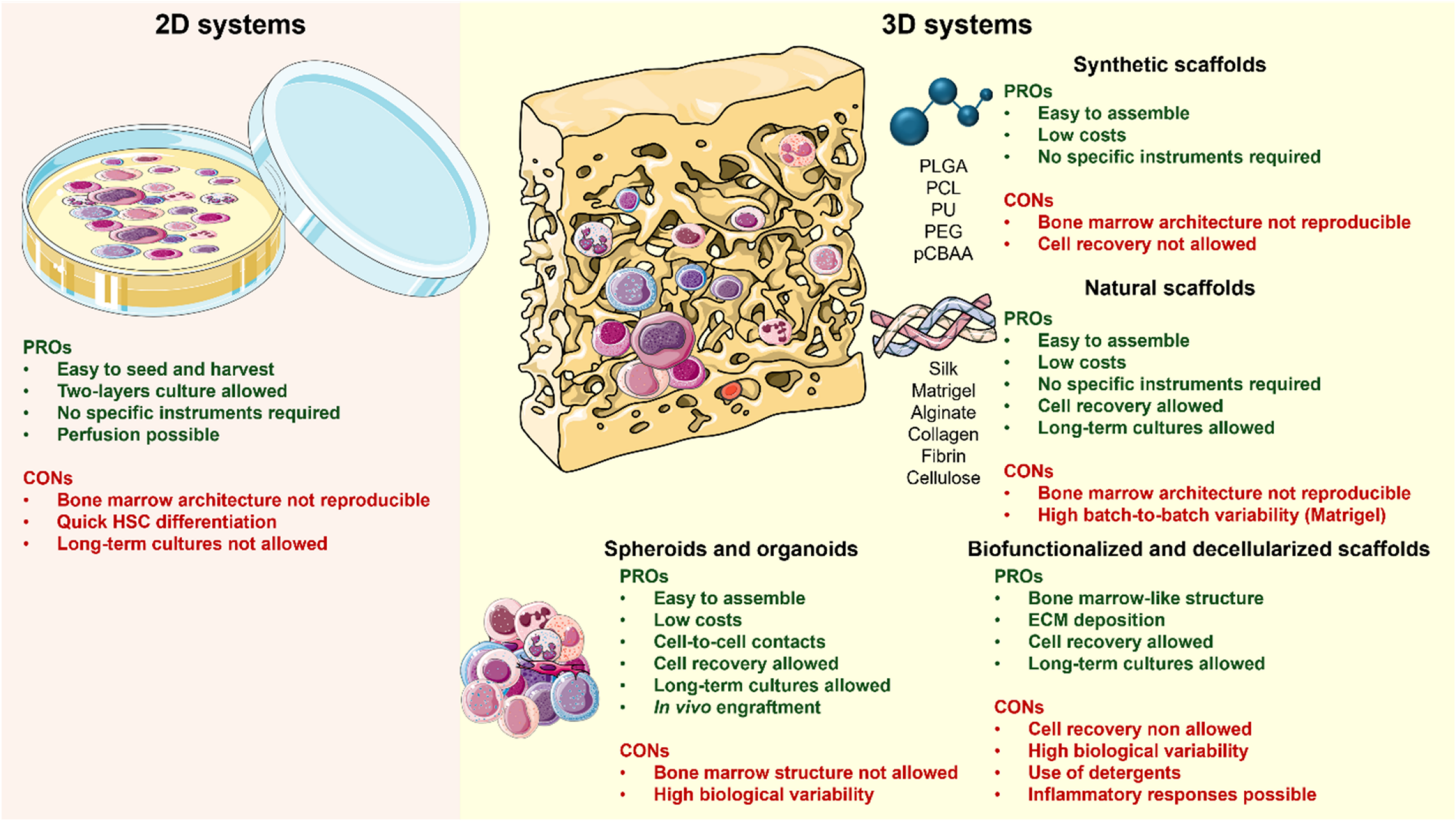
Summary of advantages and disadvantages of 2D culture systems and 3D *in vitro* models for bone marrow niche reproduction. In the left panel, 2D culture systems of hematopoietic stem and progenitor cells are carried out using standard well/Petri dishes with various mixtures of growth factors and cytokines, and the pros and cons of this model are displayed. On the right panel, a representation of the complex in a 3D *in vitro* system compared to standard 2D cultures, as the 3D model should include a supporting matrix where cells can allocate, proliferate, and differentiate into various cell types. For each supporting material and 3D-culture method, the pros and cons are presented. Created using https://smart.servier.com/.

**Table 1:** 3D Bone Marrow Niche Models^a^

materials	advantages	disadvantages	refs
Synthetic Scaffolds			
PLGA	Biocompatible and biodegradable	Not degradable for cell recovery	^96^
	Easy to produce	Bone marrow architecture not reproducible	
	Good HSC expansion		
PCL	Elastic		^96^
	Slow degradation rate		
	CD34^+^ adhesion and proliferation		
PU	Biocompatible		^97^
	CD34^+^ proliferation, differentiation and egress		
pCBAA	Metalloproteinase-cleaveable		^90^
	HSC quiescence and expansion		
Zwitterionic hydrogels	High hydrophilicity, ionic conductivity, antifouling/antiadhesive abilities		^99^
	Nonspecific protein adsorption resistance		
	Increased biocompatibility		
Matrigel	High proliferation of CD34^+^CD38^+^ cells	Variable batch-to-batch composition	^104^
	Longer stemness maintenance	Nonhuman origin	
	F-actin positive stress fiber production	Low reproducibility	
		Bone marrow architecture not reproducible	
PEG	HSC stemness maintenance over time	Not degradable for cell recovery	^98^
	Proliferation and differentiation potential into various lineages	Bone marrow architecture not reproducible	
Silk	RBCs cluster in EBI-like structures	Not degradable for cell recovery	^113^
	Differentiation toward mature CD71^+^CD235^+^ phenotype	Requires *Bombyx mori* cocoons culture and expansion	
GelMA/GelMAL	Easy to assemble	Low expansion and stemness maintenance	^104^
			^117^
			^118^
			^120^
TrueGel3D (Sigma-Aldrich)	Cell phenotype maintenance up to 21 days of culture	Variable cell recovery rate	
Biofunctionalized Scaffolds			
Ceramic Scaffolds + MSCs + Osteoblasts	ECM deposition	Not degradable for cell recovery	^122^
	Cytokine production		
	HSC expansion		
PEG hydrogel + RGD peptide	HSC expansion		^90^
	Stemness maintenance		
Gelatin-based porous scaffold stromal cells	HSC expansion		^112^
	Stemness maintenance		
Bioderived bone scaffolds + MSCs + Osteoblasts	HSC adhesion and expansion		^111^
	Stemness maintenance		
Natural Materials			
Alginate	Long-term HSC stemness	Bone marrow architecture not reproducible	^13^
	Self-renewal and differentiation	Variable cell recovery rate	^101^
			^104^
Collagen	Biodegradable		^96^
	Elastic		
	HSC expansion		
Fibrin	Stemness maintenance		^96^
	Morphological, migratory, and adhesive properties		
	Highest engraftment number		
Cellulose	Abundant and low-cost material	Not biodegradable	^90^
		Not support CD34+ cell growth	
Decellularized 3D Scaffolds			
	Native architecture and composition preservation	Detergent use for decellularization	^137–140^
	Reduced immune response to extracellular matrix components	High pressurized systems for decellularization	
	HSC expansion	Residues can trigger inflammation	
Spheroids and Organoids			
Spheroids	Successfully adult stem cells expansion	Bone marrow architecture not reproducible	^143–150^
	HSC organization in hematospheres		
	Enhanced *in vivo* engraftment		
Collagen microspheres+ MSCs	HSC and MSC proliferation		
Mesenspheres	HSC expansion		
Cord blood fibroblast organoids	Chondroid scaffold production in the mouse model	Bone marrow architecture not reproducible	
		No studies in human	
Organ-On-A-Chip			
	Bone marrow microenvironments characteristics mimicking		^162–171^

a **Abbreviations**: PLGA, poly lactic-co-glycolic acid; HSC, hematopoietic stem cell; PCL, polycaprolactone; PU, polyurethane; pCBAA, polycarboxybetaine acrylamide; PEG, polyethylene glycol; RBCs, red blood cells; EBI, erythroblastic island; MSCs, mesenchymal stem cells; ECM, extracellular matrix.

**Table 2:** Comparisons between 3D Models^a^

model	species	cell sources	expansion fold	stemness maintenance	culture time	refs
PCL	Human	UCB CD34^+^ cells	880	++	10 days	^96^
PLGA		UCB-MSCs	7.1	+		
Fibrin			5 × 10^8^	++++		
Collagen			10^4^	+++		
PU	Human	Healthy PBMCs	N.e.	+	28 days	^97^
PEG	Human	UCB-HSCs	4.9 or 12.7	+ or + ++	10 day	^98^
		UCB- or BM-MSCs				
Zwitterionic	Human	UCB	322	76.2% HSCs	14 + 10 days	^99^
Calcium chloride + gelatin + sodium alginate	Human	BM-MSCs	∼30	60.2% HSCs	7 days	^101^
GelMA	Human	UCB-HSCs + BM-MSCs	∼50% viable cells	N.e.	10 days	^104^
	Mouse	HSCs	∼7	∼2–7% HSCs	7 days	^117^
	Mouse	HSCs	2–3	∼1–14% HSCs	7 days	^118^
GelMAL	Mouse	HSPCs	1.5–10	N.e.	7 days	^120^
Alginate	Human	UCB-HSCs + BM-MSCs	∼4 × 10^4^	+	10 days	^104^
	Human	PBSCs + BM-MSCs	N.e.	∼1–3% CD34+ cells	21 days	^13^
Matrigel	Human	UCB-HSCs + BM-MSCs	∼6 × 10^4^	++++	10 days	^104^
PuraMatrix	Human	HSPCs + BM-MSCs	10–50	Differentiation after 6w	6 weeks	^107^
	Human	BM-MNCs	3	∼15% HSCs	7 days	^108^
BDBS	Human	UCB-HSCs + BM-MSCs	8	6% HSCs	10 + 14 days	^111^
Silk	Human	HSPCs	N.e.	10–80% EBI-like	21 days	^113^
Ceramic	Human	UCB-MSCs	<5	<5 × 10^5^ cells	7 days	^122^

a *Abbreviations*: PCL, polycaprolactone; PLGA, poly lactic-co-glycolic acid; PU, polyurethane; PEG, polyethylene glycol; GelMA, Gelatin methacryloyl; GelMAL, maleimide-modified gelatin; BDBS, bioderived bone scaffolds; UCB, umbilical cord blood; MSCs, mesenchymal stem cells; PBMCs, peripheral blood mononuclear cells; HSCs, hematopoietic stem cells; BM, bone marrow; PBSCs, peripheral blood stem cells; BM-MNCs, bone marrow mononuclear cells; EBI, erythroblastic island; MSCs, mesenchymal stem cells; N.e., not evaluable.

### 3.1 Scaffold-Based 3D Models

Different types of materials have been tested to build the most efficient scaffold system for 3D bone marrow niche model, and they can be natural, synthetic, biodegradable, and biocompatible.^90^ A range of materials is used to produce artificial scaffolds, typically those porous polymers that support cell infiltration and growth. These materials are widely used because of the low costs, they are easy to manage and assemble, and they do not require specific instrumentation; however, these scaffolds have a simple texture and lack the complexity of extracellular bone marrow matrix. Conversely, natural scaffolds from decellularized tissues preserve the 3D structure, offer low cytotoxicity and immunogenicity, and have a high repopulation potential.^92^ Scaffold-based models have been used for over a decade for *in vivo* hematopoietic regeneration, as they form a 3D reticular structure where cells can locate and, through nutrients and molecules, can freely circulate, allowing good exchanges also within the core of the scaffold.^93^ Biomaterials can mimic the ECM and provide a biological platform for cell-ECM interaction, mechanotransduction, and extracellular signaling. The presence of pores within the scaffold reduces reactive oxygen species (ROS) production compared to nonporous systems, and matrix stiffness also influences *in vitro* hematopoiesis. Moreover, multiple hydrogels are translucid, and cells can be directly observed by optical microscopy.^94^ In Table 2, expansion fold, viability percentages, duration of HSC stemness maintenance, and other characteristics of scaffold-based 3D bone marrow niche systems are reported for comparison.^95^

3D-culture approaches using both synthetic and natural polymers can preserve *ex vivo* HSC characteristics compared to 2D culture. Among available polymers, such as polycaprolactone (PCL), poly lactic-co-glycolic acid (PLGA), fibrin, and collagen, PLGA can better support HSC expansion, while fibrin can longer maintain stemness phenotype,^96^ as well as porous polyurethane (PU) scaffolds.^97^ Integrin anchorage peptides,^98^ cleavable sites for biodegradation,^72^ and newer biocompatible Zwitterionic hydrogels have also been developed.^99^ Zwitterionic hydrogels are formed by molecules with both cationic and anionic groups, resulting in a neutrally charged structure. This unique characteristic exerts several advantageous properties, including high hydrophilicity, ionic conductivity, antifouling/antiadhesive abilities, and resistance to nonspecific protein adsorption (nonfouling), that can trigger *in vivo* coagulation and inflammation, thus increasing biocompatibility.^100^

Alginate is a negatively charged, hydrophilic, and noncell-adhesive polysaccharide, which in the presence of calcium cations immediately gels, while the removal of cations with calcium chelators can dissolve alginate gel instantly. Alginate gel has a porous structure that favors solute permeability, high water content, and mechanical properties resembling those of biological tissues. Within alginate, HSCs assume a spherical shape, proliferate and maintain their CD34^+^ stemness phenotype.^13,101^ 3D coculture using alginate-based hydrogels with bone marrow mononuclear cells/peripheral blood stem cells and BM-MSCs can successfully maintain HSC stemness and differentiation capacity for up to 21 days. Alginate hydrogels allow efficient and easy assembly and can be perfused by assuring oxygen and nutrient exchange also at the core and quick recovery of live cells for further analysis. Alginate beads resemble spheroids, where alginate polymers offer structural support for cells and allow cell-to-cell communications and paracrine signals. For these reasons, alginate beads can be cultured in media without cytokines or growth factor supplements, while still maintaining long-term HSC stemness, facilitating self-renewal and differentiation.^13^

Matrigel, a widely used cell culture matrix, is mainly composed of laminin (∼60% of total components) and COL4 (∼30%), while entactin allows one to link these two proteins. Heparan sulfate proteoglycans increase cell adhesion. Matrigel also contains other various components, such as growth factors like TGF-β, epidermal growth factor, platelet-derived growth factor, collagenases, plasminogen activators, and other undefined proteins.^102^ Because of its composition, Matrigel closely resembles basement membrane composition and can be used for 3D cell cultures and tissue regeneration.^103^ Indeed, Matrigel has been employed for *in vitro* bone marrow models, resulting in a higher proliferation of CD34^+^CD38^+^ hematopoietic cells compared to alginate-based cultures.^104^ Moreover, CD34^+^CD38^–^ populations cultured in Matrigel longer maintain their stemness phenotype longer than alginate, and stromal cells form dense networks of cadherin-mediated intercellular junctions, resembling marrow architecture.^105^ Cells cultured in 2D conditions display a rounded morphology, whereas when cultured in 3D systems, cells assume specific morphologies and produce F-actin^+^ stress fibers, important for tissue remodeling and differentiation.^106^

The PuraMatrix hydrogel (Corning) consists of amino acids (1% w/v) and 99% water and has been successfully employed for HSC expansion starting from peripheral blood mononuclear cells, while the methacrylate hydrogel has been used in mouse-derived HSCs cocultured with MSCs.^107,108^

Polyethylene glycol (PEG) porous scaffold can be employed for 3D bone marrow cultures, as HSC stemness is maintained over time in a coculture system of bone marrow mesenchymal stem cells and osteoblasts without compromising their potential to proliferate and differentiate into various lineages.^109^ PEG can also be used for the production of hydrogels functionalized with adhesive motifs, such as the arginyl-glycyl-aspartic acid (RGD), which has also been used to functionalize other scaffolds, like the TrueGel3D (Sigma-Aldrich).^110^

A different approach is to culture stromal cells, such as MSCs, osteoblasts, and endothelial cells, on a prefabricated scaffold, because these cells can produce and release natural extracellular matrix components, that deposit on the scaffold, thus creating a highly biomimetic platform for HSC culture.^111,112^ In this type of system, HSCs tend to remain anchored to the scaffold, while differentiated cells are more likely to be released in the culture medium.

Silk-based scaffold is a promising system for reproducing the *in vitro* bone marrow niche. Silk fibroin produced by native *B. mori* is composed of heavy and light chains linked by disulfide bonds, with a structure rich in hydrophobic β-sheet regions that provide high strength and toughness. Silk is also biodegradable, and its degradation influences cell metabolism and osteogenesis. Silk fibers are already employed in surgical sutures, where they are wax-coated to reduce fraying and immune response, which is similar to that elicited by collagen and PLA. Silk fibroin 3D scaffolds have been used for *in vitro* production of erythrocytes. In this system, fibronectin creates a net within erythroblastic island­(EBI)-like niches, where red blood cells (RBCs) uniformly distribute and cluster in EBI-like structures. Moreover, as the culture proceeds, RBCs differentiate from a CD71^+^CD235^–^ phenotype to a mature CD71^+^CD235^+^ phenotype, favored by cocultures with macrophages.^113^

Scaffold-based 3D bone marrow niche can also be obtained using prefabricated hydrogels, such as TrueGel3D (Sigma-Aldrich) prepared by combining nonanimal polymers with cross-linkers. This hydrogel maintains cell viability and reproduces the natural extracellular matrix network, supporting cell adhesion and migration in environments similar to those of tissues. Basic components are dextran, or poly­(vinyl alcohol) (PVA), and PEG or cyclodextrin with thiol functionality, which are used as cross-linkers to connect polymer chains and form the transparent hydrogel. Indeed, replacement of serum albumin with PVA is associated with ex vivo expansion of functional HSCs using limiting dilution transplantation assays and split-clone transplantation methods.^114^ Other molecules to improve matrix adhesion can also be embedded within this scaffold, such as the TrueGel3D Arg-Gly-Asp (RGD) integrin adhesion peptide, fibronectin, laminin, peptides, heparan sulfate, or growth factors. Moreover, cells can be recovered after culture using a nontoxic degradation enzyme, and cells can be used for further experiments and characterization. Our preliminary data showed that TrueGel3D could be used to set up a 3D *in vitro* coculture model of leukemic bone marrow, by embedding Wharton’s Jelly-derived MSCs from healthy donors and a hairy cell leukemia (HCL) cell line (BONNA-12, DSMZ) within the prefabricated scaffold. Briefly, once expanded, MSCs were seeded at various ratios with HCL cells (1:1 or 1:2) and at various densities (2.5 × 10^5^ cells/mL, 5 × 10^5^ cells/mL, or 1 × 10^6^ cells/mL) using the biomimetic scaffold TrueGel3D Hydrogel (Sigma-Aldrich). First, cells were mixed with the hydrogel, and the mixture was allowed to polymerize in the presence of the cross-linker, following the manufacturer’s instructions. Scaffolds were then cultured in α-MEM (STEMCELL Technologies) supplemented with 100 nM dexamethasone (Sigma-Aldrich), and cultured at 37 °C in an atmosphere of 5% CO_2_ and 95% relative humidity up to 21 days. At 7, 14, and 21 days, scaffolds were disassembled using the TrueGel3D Enzymatic Cell Recovery Solution (Sigma-Aldrich), cells were recovered, washed, and labeled with anti-CD90 FITC-conjugated antibody for identification of MSCs, and samples were acquired on a FACSVerse cytometer to confirm the presence of both cell populations at the end of culture. MSCs and leukemic cells were present and viable, showing the potential of this scaffold-based 3D system as a suitable method for a 3D *in vitro* coculture system of leukemia.

Another prefabricated scaffold for the 3D *in vitro* bone marrow model is GelMA, composed of gelatin (Gel) and methacrylic anhydride (MA). Gelatin can be porcine or bovine with bioactive motifs, a low melting point, can dissolve at body temperature, and is functionalized to cross-link under UV or visible light irradiation in the presence of photoinitiators.^115^ After cross-linkage, GelMA forms a stable hydrophilic 3D polymerized network, which is highly permeable for the exchange of water, nutrients, and metabolic wastes.^116^ This system has been employed for HSC culturing also by using a covalently immobilized SCF-functionalized GelMA hydrogel;^117,118^ however, UV photo-cross-linking could negatively affect cell viability and DNA integrity.^119^ For this reason, a maleimide-functionalized gelatin (GelMAL) chemically cross-linked with 1,4-dithiothreitol (DTT) has been optimized for HSC encapsulation in large macrogels.^120^

Biomaterials can be mixed to form hierarchical structures and to obtain more complex systems, resembling organ-like models, like interleaved lattice-mesh PCL structure spiked with hydroxyapatite (lattice) and polyurethane (mesh) coated with vitronectin and used to expand umbilical cord blood-derived HSCs.^121^

Ceramic-based scaffolds can highly mimic the trabecular bone structure and are able to sustain osteogenic differentiation of MSCs and matrix deposition in a 3-week culture. These engineered niches have also been injected into mice, and HSC expansion and differentiation have been observed.^122^

Hybrid or conventional double-network (DN) hydrogels are of growing interest in the tissue engineering field, and hybrid DN hydrogels are composed of one covalently or ionically cross-linked synthetic polymer and a second ionically cross-linked natural polymer. The choice of polymers used is based on toughness, stretchability, or other physical properties required;^123^ however, DN hydrogels show poor degradability, cell infiltration, and ECM deposition, due to the lack of interconnected macroporous structures.^124^ Conversely, cryogels possess these features, as gel precursors are cross-linked at subzero temperatures, where most solvents freeze, while gel precursors cryoconcentrate in small volumes, which accelerate polymerization or gelation.^125^ Cryogels have multiple advantages over other matrixes, such as increased elasticity, quick swelling kinetics, high compression loads, physical resistance, high water uptake and mass transfer of solutes, optimal cell infiltration, and long-term cell culture.^126^ Cryogels can be made by several materials, such as alginate and collagens, and bone marrow cryogel (BMC) releasing bone morphogenetic protein 2 can enhance T cell differentiation and HSC engraftment in mouse models.^127^

Scaffold-based approaches are widely utilized as 3D *in vitro* bone marrow models, as some of them are easy to assemble and have low costs, like collagen- and alginate-based platforms; however, in these systems, cells are mixed with molecules, and mixtures are allowed to jellify or polymerize. Therefore, embedded cells do not distribute with a precise architecture, thus these types of scaffolds do not mimic normal bone marrow structure. Conversely, these scaffolds can be quickly disassembled, using appropriate degradation enzymes (e.g., collagenases) or ion chelators (e.g., EDTA), cells can be recovered, and used for further characterization and/or experiments. Cell recovery rates might vary depending on the used materials, and they can be low, especially when MSCs are cocultured, because of matrix deposition. Pure synthetic scaffolds, like those made with PLGA, PLC, PU, or Zwitterionic polymers, are easy to assemble, are chip materials, and are less immunogenic, as they do not carry potential antigens that could trigger immune responses and inflammation. For these reasons, these scaffolds offer neutral support for HSC proliferation and expansion, although they do not allow them to reproduce in vitro the normal bone marrow architecture. In addition, these materials could not be easily disassembled, and cells can not be recovered for further processing [12–13.95].

Matrigel is the most similar to human basement membranes; however, its composition can greatly vary from batch to batch, thus reducing large-scale experimental reproducibility, especially if these systems could be translated into clinical practice. As other scaffolds, Matrigel is a culture matrix, and cells are just mixed together; thus, they do not assume precise locations, as in normal bone marrow structure. Similarly, GelMA and GelMAL have the same cons, plus they require cross-linkage with UV or DTT. Silk-based scaffolds are the most promising platforms, although their production still requires specific instruments and expertise for *Bombyx mori cocoons*.^119,120^

Preliminary cocultures of MSCs, osteoblasts, and endothelial cells allow the production of prefabricated scaffolds that closely resemble the normal bone marrow architecture, in which HSCs can be seeded. However, cells can not be recovered for further experiments, similar to that reported when cells are seeded in hydroxyapatite-based scaffolds, where scaffold disruption is challenging. In addition, with this latter platform, although it closely mimics bone tissue, its production requires various chemicals and specific instruments for scaffold lyophilization.^12^

### 3.2 Decellularized 3D Models

Decellularized 3D scaffolds are natural biomimetic platforms produced from decellularized tissues obtained from various sources.^128,129^ In this approach, we take advantage of already structured physiological extracellular matrix and tissue architectures, that closely resemble normal cellular environments and are currently used as platforms for whole organ engineering.^130^ These scaffolds can be obtained from several species, such as pigs, cows, horses, and humans.^90^ In comparison to synthetic scaffolds, natural decellularized scaffolds preserve native architecture and composition, which mediate cellular responses. These characteristics support integration into host tissue after implantation and may result in a reduced immune response to extracellular matrix components.^131^ Complete removal of cells and residual DNA is necessary to prevent unwanted immune reactions, particularly with scaffolds from nonhuman species, and physical methods that induce cell lysis, such as freeze/thaw cycles, are commonly used.^90,132^ However, precise temperature control during freeze–thaw cycles is required to protect the ultrastructure of the extracellular matrix, and freeze–thaw cycles alone do not remove all cellular content. Therefore, additional chemical and enzymatic treatments are necessary, including the use of detergents and/or enzymatic digestion or combinations of salt solution rinses with enzymatic or detergent rinses. Sodium dodecyl sulfate (SDS) is often employed for larger tissue decellularization where milder detergents are less effective; however, SDS may damage vascular structures, and some residues could persist, resulting in impaired DNA and RNA enzymatic digestion and cellular viability.^133^ Moreover, some detergents could affect scaffold biomechanical properties.^134^ For these reasons, detergent-free decellularization methods are being investigated, such as repeated freeze/thaw cycles or high hydrostatic pressure.^135^ This method does not require comprehensive prior knowledge of all niche elements and specific equipment, while preserving the chemical and physical characteristics of natural tissue. However, decellularized scaffold composition is a high variable, thus representing a bias for reproducibility and reliability of large-scale experiments. For example, scaffold components may vary based on the bone areas where they are obtained, as the endosteal region and the central bone area are physiologically different.^136^

Decellularized matrices can be obtained using poly­(methyl methacrylate) templating of poly­(ethylene glycol)-diacrylate-co-N-acryloyl 6-aminocaproic acid (A6ACA) (PEGDA-co-A6ACA). This dual-component matrix shows an inner nonmineralized compartment and an outer mineralized section, simulating the long bone tissue architecture, allowing proliferation and osteogenic differentiation of both mouse and human mesenchymal stromal cells cultured within the mineralized part, while not in the inner compartment, which retains a higher percentage of human CD34^+^ HSCs up to 14 days of culture.^137^

Decellularized matrices have been employed to mimic human bone marrow tissues and to study the effects of cosmic radiation on hematopoiesis, by first culturing for 4 weeks induced pluripotent stem cells-derived or bone marrow-derived MSCs into decellularized bone matrix scaffolds, then by infusing MSCs and human umbilical vein endothelial cells mixed with fibrin hydrogels into the new bone, and subsequently by adding cord blood-derived CD34^+^ HSPCs, for 1–2 week culture.^138,139^ This protocol has also been employed to reproduce tumor-induced metastatic colonization of human bone marrow and to study pathological mechanisms of tumor metastasis and microenvironment.^140^ Similarly, to study tumor growth and progression within the bone marrow of myeloid neoplasms, an alternate-direction perfusion system composed by pores of 3D hydroxyapatite scaffolds and bone marrow-derived MSCs can be used for culturing umbilical cord blood HSPCs or human primary CD34^+^ cells derived from myelodysplastic patients, for a total of 3-week period.^141^ BM-derived MSCs can also be seeded in ceramic materials within a perfusion bioreactor,^142^ or by first bioprinting a decellularized matrix of β-tricalcium phosphate bioceramics.^143^

Decellularized ECM (dECM) is obtained from human BM stromal cell line, the HS5, from traditional 2D cultures, or from spin-coated dECM.^144^ Each of these dECM has peculiar characteristics, as spin-coated dECM has a more uniform distribution, double roughness, and higher HSC expansion rates. Other sources are the decellularized Wharton jelly matrix (DWJM) as an ECM scaffold used to embed human BM-MSCs as supporting niche cells. This model has been tested for HSC expansion by seeding umbilical cord blood-derived HSCs, showing optimal proliferation, viability, self-renewal and differentiation abilities.^145^ Another decellularized 3D bone marrow model example is the natural scaffold from decellularized bovine bone marrow with conserved native 3D-architecture, including blood vessels, cell niches, and ECM compositions with COL3, COL4, and fibronectin. This decellularized structure can be seeded with HS5-derived stromal cells and umbilical cord blood-derived HSCs, demonstrating a good proliferation rate and viability.^146,147^

Decellularized 3D models take advantage of normal tissue structures, as nonhuman tissues are deprived of cells and residual nucleic acids, and human cells are seeded within, and relocate according to their normal spatial distribution in the bone marrow. However, the decellularization process is complex and requires detergents that can be toxic to cells or highly pressurized systems that are not widely available. Residual cells and nucleic acids can trigger immune responses and inflammation, thus altering experimental results. Moreover, cells can not be recovered for further investigation.

### 3.3 Spheroids and Organoids

Spheroids and organoids are 3D cell structures that differ in complexity and formation, as spheroids are self-assembled cell aggregates forming microspheres without a scaffold support, while organoids are made from organ-specific stem or progenitor cells, require scaffolds, and grow into miniature organs suitable for advanced 3D studies. Spheroid cultures have successfully been used to expand various adult stem cells,^148^ such as MSCs, that differentiate toward osteoblasts and deposit extracellular bone matrix, which can be further used as a decellularized scaffold.^149^ Spheroids have also been employed for the HSC culture, and for HSC expansion starting from peripheral blood mononuclear cells with minimal HSC frequency, as they can self-organize in hematospheres under nonadherent conditions.^150^ Nestin^+^ MSCs have been used to produce mesenspheres for HSC maintenance, self-renewal, and multipotency, and HSCs cocultured with mesenspheres show expanded transplantable potential and enhanced *in vivo* engraftment.^151^ In addition, cord blood fibroblast pellets can be cultured and differentiated into cartilaginous tissue *in vitro* and then implanted in mice, resulting in ossicles with bone marrow architecture, vascular structures resembling sinusoids, and hematopoietic tissues after 8 weeks. These organoids can support human HSC engraftment and hematopoiesis *in vivo*.^152^ COL1/matrigel matrix has been used in a bone marrow-like organoid (BMO) system, where embryoid bodies have been embedded in and triggered to differentiate toward HSPCs upon chemical stimuli. Starting from day 17 of culture, BMO cells successfully differentiate into CD45^-^CD31^+^ endothelial cells, CD45^+^CD11b^–^CD34^+^ HSPCs, CD45^+^CD11b^+^ myeloid cells, CD45^–^CD31^–^CD271^+^CD90^+^CD105^+^CD73^+^ mesenchymal stem/progenitor cells, and CD45^–^CD31^–^CD271^+^ mesenchymal stromal cells, spatially organized resembling hematopoietic niche architecture.^153^ COL1/matrigel matrix can also be used for induced pluripotent stem cell (iPSC) organoids, where cells successfully differentiate into vascular and hematopoietic cells, mimicking the bone marrow niche with a well-vascularized architecture. Upon appropriate stimulation, organoids can reproduce a pro-fibrotic environment, like that observed in myeloproliferative neoplasms, or they can favor *ex vivo* viability maintenance of multiple myeloma cells through cell contacts with other stromal cells and to matrix support.^154,155^ Human iPSC-derived bone marrow organoids have also been employed for mimicking myelodysplastic marrow, and seeded HSPCs successfully migrate within organoids, maintain self-renewal abilities, their genetic profiles, and their different phenotype compared to normal hematopoietic cells.^156,157^ Human iPSCs can be cultured and expanded in Matrigel with mTeSR plus medium, and obtained cells can be seeded in ultralow attachment (ULA) plates to form embryo bodies and stimulated with bone morphogenetic protein 4 (BMP4), VEGFA, fibroblast growth factor 2 (FGF2) at 25 ng/mL, and IL-21 ligand at 5 ng/mL under hypoxia condition (1% O_2_) for 72 h for mesoderm formation and angiogenesis induction. Next, embryo bodies can be stimulated under normoxia for 2 days and SCF and FLT3 at 25 ng/mL for hemogenic endothelium induction. These formed bodies can be embedded in a hydrogel composed of Geltrex (Gibco), VitroCol (Advanced Biomatrix), and COL4 (Advanced Biomatrix) for solidification, in APEL2 medium supplemented with VEGFA, VEGFC, FGF2, BMP4, FLT3, SCF, granulocyte colony-stimulating factor (G-CSF), TPO, EPO at 50 ng/mL, IL-3, and IL-6 at 20 ng/mL, and embryo bodies mature into self-assembling 3D structured organoids over a 7-day hydrogel culture.^156^ In another protocol, after embryoid body (EB) formation in a COL1/matrigel matrix, mesoderm can be first induced by Wnt agonist CHIR99021, BMP4, and VEGF stimulation (at day 0), and next hemogenic endothelium using the activin/nodal pathway inhibitor SB431542, bFGF, SCF and VEGF (at day 2), while hematopoietic progenitor cells are induced on day 4 using cytokines and vascular structures employing low dose VEGF from day 8 until day 10, when embryoid bodies can be transferred to ULA plates to promote organoid maturation.^157^

Organoids can support survival and proliferation of primary multiple myeloma, acute lymphoblastic leukemia (ALL), and Xeno iALL cells, with improved survival compared with standard 2D conditions or a single-lineage 3D coculture system containing primary human bone marrow MSCs in a Matrigel + COL1 hydrogel. Primary neoplastic plasma cells in organoids show minimal proliferation; however, aberrant CD38^+^CD319^+^CD56^+^ immunophenotype is retained, as well as ALL cells with maintenance of CD19 expression.^158^ Acute myeloid leukemia (AML) organoids have also been assembled starting from NSG mice engrafted with human primary AML cells and seeded into polyurethane scaffolds in serum- and cytokine-free medium. CD33^+^CD44^+^ AML cells are viable and expand from 7% at day 0 to >10% at day 70 of culture, and they rearrange the typical leukemic niche within organoids, including expression of fibronectin, VCAM-1, and N-Cadherin, surrounded by Osteopontin- and Osterix-expressing cells.^159^ Moreover, AML cells from day 70 organoids retain the ability to re-expand and form secondary organoids with long-term culture properties.^159^

Spheroids and organoids are the simplest models in which cells can communicate through cell-to-cell contacts and by paracrine signals, and they can be disassembled. However, bone marrow architecture is not reproduced; thus, these systems are not appropriate to study normal marrow structures and functions.

### 3.4 3D Printing

Recent developments in 3D bioprinting and biomimetic materials have allowed the generation of complex living tissue constructs using biomaterials and cells. New 3D bioprinting techniques have produced reconstructions of intricate tissue structures using hydrogels, collagen, and other materials.^160^ 3D bioprinting presents significant potential for 3D stem cell culture, tissue engineering, and clinical applications, though technical challenges remain, including resolution for microscopic structures, limitations in replicating vascular networks, gelation issues, and cellular viability.^161^

Some HSC 3D-culture approaches use 3D printing to emulate the bone marrow niche. For example, a 3D printed hydrogel mesh loaded with MSCs supported HSC proliferation and was effective as a coculture scaffold, outperforming conventional 2D coculture in HSC expansion.^162^ This 3D scaffold, however, did not mimic the bone marrow niche’s anatomical structure. A more advanced approach used a two-compartment model: a 3D printed calcium phosphate cement (CPC) scaffold seeded with MSCs, differentiated to osteoblasts to replicate the endosteal niche, and a Matrigel containing endothelial and MSCs to emulate the perivascular niche was integrated into the CPC scaffold, enabling cell interaction and migration between compartments. This system supports CD138^+^ primary myeloma cell proliferation and serves as a model for myeloma pathophysiology, although its application for HSC culture has not been evaluated.^163^

3D bioprinting is the newest and most promising approach, and it could be successfully combined with organ-on-chip methods, as bone marrow-like chips can be 3D bioprinted and used to coculture different types of cells under perfusion. Moreover, chips with different conditions (e.g., resembling different organs) can be connected, and interactions between systems can also be explored. In these platforms, a cell culture matrix can also be employed, to better mimic bone marrow extracellular matrix composition.^12^

### 3.5 Organ-on-Chip

Recent advances in 3D *in vitro* models have improved basic research and drug screening by simulating complex tissue microenvironments and reducing the reliance on animal models. A combination of tissue engineering and microfluidics has created the “organ-on-a-chip” systems, which are 3D *in vitro* human tissues.^164,165^ Microfluidic devices can replicate some characteristics of bone marrow microenvironments but miss aspects like cell egress into the bloodstream.^166^ Developed bone marrow organ-on-a-chip models each have limitations, as some authors have employed mouse cells and in vivo culture, while others have incorporated human cells but lacked vascular models, or have included a bone scaffold with endothelial cells and mesenchymal stem cells, forming basic vessels but not demonstrating hematopoiesis, or have not maintained long-term stemness.^167,168^ A microfluidic device consisting of two hexagonal chambers connected by three symmetric two-way ports has been designed based on the capillary burst valve concept for sequential loading of fibrin hydrogels restricted to each adjacent chamber but still permitting diffusion of soluble signaling molecules and migration of cells. A third bottom chamber adjacent to the hexagonal chambers can be used to load a third cell type at a later time point. This platform, under 1 mm, features a 3D perfusable vascular network, distinct perivascular and endosteal niches, hematopoiesis, neutrophil migration, progenitor maintenance with CFU-GEMM formation, and breast cancer cell migration into the niche, over a 14-day coculture period.^169^ However, the small chamber volume allows seeding of a limited number of HSCs, making high-throughput experiments challenging. Moreover, although more similar to the physiological bone marrow niche, organ-on-chip models are designed by selecting which organ-specific features need to be included. For instance, bone innervation, which influences bone development and remodeling, is frequently omitted, mostly to simplify models.^90^ Fibrin gel embedded with human CD34^+^ cells, BM-MSCs, and human umbilical vein endothelial cells (HUVECs) in a 2-channel microfluidic device allows HSC proliferation and differentiation toward myeloid and erythroid lineage upon cytokine stimulation, and spontaneous migration toward the vascular channel resembling the intravasated phenomenon. This system has also been used as a human *in vitro* preclinical model to predict hematological toxicity of mostly used drug exposure, or to mimic bone marrow defects in the Shwachman–Diamond Syndrome.^170^ A multicompartment model can be achieved using a multichannel organ-on-chip device that can incorporate the endosteal, MSCs, and perivascular niche. In this multiniche device, MSCs differentiate toward osteogenic lineage and support mineralization, thus reproducing a bone-like layer that can host HSCs, endothelial cells, and MSCs in fibrin-collagen material.^171^ Pathological bone marrow niche conditions can also be represented in an organ-on-chip model. In particular, B cell B-ALL in vitro organotypic “leukemia-on-a-chip” model has been developed to investigate chemotherapy resistance, showing that leukemic perivascular, endosteal, and hematopoietic niche-derived factors promote B-ALL cell survival and quiescence, as described *ex vivo* and in other models.^172^ This leukemia-on-a-chip model can be fabricated using standard soft lithography with polydimethylsiloxane (PDMS), and cell culture carried out using a compartmentalized system composed of a central venous sinus, a medullary cavity, and endosteal regions connected with four medium reservoirs for long-term medium supply and partitioned by regularly spaced trapezoid micropillars.^172^ This leukemia-on-a-chip model has also been employed to study spatiotemporal monitoring of chimeric antigen receptor (CAR)-T cell extravasation, recognition of leukemic cells, immune activation, cytotoxicity, and tumor killing abilities. Moreover, this system can be used in clinical practice to predict responsiveness to CAR-T therapies,^173^ also in multiple myeloma, termed Multiple Myeloma-on-Chip (MMOC) with eight channels with individual inlet and outlet ports for media flow and introduction of immune cells and CAR-T lymphocytes.^174–177^ Moreover, using these approaches, the MM-associated niche can be studied, as previous reports have observed altered EC organization, wide EC junction pores with increased permeability.^176^ Organ-on-a-chip models in hematology have also been implemented for the investigation of lymphomas, by resembling the lymph node structure.^177^ Although outside the scope of this review, it is worth mentioning the lymphoma-on-chip model, optimized for the investigation of diffuse large B cell lymphoma cells within their microenvironment, composed of lymph node stromal cells, fibroblastic reticular cells, and lymphatic endothelial cells. In particular, lymphatic endothelial cells coat a tubular vessel, which is surrounded by a hydrogel embedded with neoplastic cells and fibroblastic reticular cells.^178^

## 4 Conclusions and Future Perspectives

The bone marrow niche is a highly complex ecosystem, where several cell types, such as stromal, hematopoietic, endothelial, and immune cells, interact through physical contact, soluble mediators, extracellular matrix-derived signals, and vesicles to sustain blood homeostasis. Conventional 2D culture systems are inadequate to reproduce this complexity, resulting in rapid differentiation of HSCs with a loss of their stemness potential. In contrast, 3D *in vitro* cultures may mimic the spatial organization, molecular gradients, and mechanical properties of the bone marrow niche, providing a more physiological backbone for basic research as well as translational applications. However, each 3D technique has its own specific strengths and limitations.^12^

Future directions in this field should focus on standardized protocols, including the optimal choice of stem cell sources, medium composition, and culture conditions, integration of vascular, adipogenic, and neuronal components, and validation with human cells to improve reproducibility and clinical relevance. These improvements will allow to transform 3D bone marrow models into powerful tools for *in vitro* studies on mechanisms of normal and pathological hematopoiesis, for *ex vivo* drug screening, or *ex vivo* expansion of transplantable HSCs.

Some Authors are already optimizing bone marrow-on-a-chip models by combining *in silico* and computational approaches to simulate the niche, to better mimic physiological conditions, helping to bridge the gap between *in vitro* and *in vivo* studies with replication of physical and biochemical properties of the niche, integration with personalized medicine strategies, and combination with potential gene editing approaches. Indeed, the development of patient-specific models could be used as an ex vivo drug screening platform, as ex vivo expansion of HSCs for transplantation purposes, or for specific gene editing, including CAR expression, subsequent cell expansion for therapeutic approaches. Multiorgan models derived from whole-body organ-on-a-chip systems will permit assessment of efficacy and systemic toxicity of drugs or cell invasiveness.

Current BM niche models show promise, but reliably expanding HSC populations without losing differentiation or engraftment capability remains difficult due to HSC heterogeneity and limited long-term-HSC proliferation, although some organ-on-a-chip or organoids systems maintain HSC proliferation capacity and stemness phenotype up to 70 days of culture. Improving our understanding of hematopoietic niches, especially fetal sources, may help develop better expansion methods and remove HLA compatibility issues related to donor-to-patient histocompatibility matches and related graft rejection. The major challenge for 3D *in vitro* bone marrow models remains the replication of the exact spatial heterogeneity and complexity of the physiological marrow niche, while organoids and organ-on-a-chip models could eventually fill this gap, although they still have high costs and technical barriers for broader adoption in research and clinical practice.

In conclusion, bone marrow models have evolved from standard 2D cell suspensions to sophisticated 3D systems and organ mimics, greatly advancing our knowledge of hematopoiesis and disease development and accelerating therapeutic advances including CAR-T cell therapies. As the field grows, these models promise improved treatment strategies and avenues for scalable long-term HSC expansion for transplantation purposes and gene editing approaches.

## Data Availability

Data are available upon request by the Authors.
